# Community-Based Approaches to Training the Future Workforce for an Aging Population in Challenging Times

**DOI:** 10.1093/geroni/igaf122.1040

**Published:** 2025-12-31

**Authors:** Iveris Martinez

**Affiliations:** California State University, Long Beach, Long Beach, California, United States

## Abstract

In challenging times where funding is uncertain and scarce, innovative strategies must be developed to train the future workforce of health and service providers to serve the growing and diverse aging population. The Center for Successful Aging (CSA) at California State University Long Beach brings together faculty, students, and community partners to develop creative solutions for diverse aging populations, serving as a leader in community engagement, workforce development, and applied research to address disparities and promote quality of life in aging. The CSA Internship Program has taken a community-based approach to providing both research and service-oriented learning opportunities for students at the CSULB College of Health and Human Services. Service-oriented internships have included support for a service provider collaborative and student-run dementia respite program. Research-oriented internships have included a masking adherence study, an adult day-care feasibility study, and a study on the experience of older adults dining out with hearing loss. To date, thirty-seven students have completed a minimum of 120 hours each for a total of 4,400 hours. By leveraging our community partnerships, CSA has provided research and service-oriented opportunities for students to build their knowledge and skills on aging and improve attitudes towards working with older adults. In this presentation, we will describe in more detail the strategies for developing unique learning experiences, student learning outcomes, and their impact on students. Through these strategies, CSA aims to empower students to become the next generation of leaders in health and human services for an aging society.

